# athisomiRDB: A comprehensive database of *Arabidopsis* isomiRs

**DOI:** 10.1093/database/baae115

**Published:** 2024-11-08

**Authors:** A T Vivek, Ajay Arya, Supriya P Swain, Shailesh Kumar

**Affiliations:** Bioinformatics Laboratory, National Institute of Plant Genome Research (NIPGR), Aruna Asaf Ali Marg, New Delhi 110067, India; Bioinformatics Laboratory, National Institute of Plant Genome Research (NIPGR), Aruna Asaf Ali Marg, New Delhi 110067, India; Bioinformatics Laboratory, National Institute of Plant Genome Research (NIPGR), Aruna Asaf Ali Marg, New Delhi 110067, India; Bioinformatics Laboratory, National Institute of Plant Genome Research (NIPGR), Aruna Asaf Ali Marg, New Delhi 110067, India

## Abstract

Several pieces of evidence challenge the traditional view of miRNAs as static molecules, revealing dynamic isomiRs originating from each miRNA precursor arm. In plants, isomiRs, which result from imprecise cleavage during pre-miRNA processing and post-transcriptional alterations, serve as crucial regulators of target microRNAs (miRNAs). Despite numerous studies on *Arabidopsis* miRNAs, the systematic identification and annotation of isomiRs across various tissues and conditions remain limited. Due to the lack of systematically collected isomiR information, we introduce the athisomiRDB database, which houses 20 764 isomiRs from *Arabidopsis* small RNA-sequencing (sRNA-seq) libraries. It comprises >2700 diverse samples and allows exploration at the sample, miRNA, or isomiR levels, offering insights into the presence or absence of isomiRs. The athisomiRDB includes exclusive and ambiguous isomiRs, each with features such as transcriptional origin, variant-containing isomiRs, and identifiers for frequent single-nucleotide polymorphisms from the 1001 Genomes Project. It also provides 3ʹ nontemplated post-transcriptional additions, isomiR–target interactions, and trait associations for each isomiR. We anticipate that athisomiRDB will play a pivotal role in unraveling the regulatory nature of the *Arabidopsis* miRNAome and enhancing sRNA research by leveraging isomiR profiles from extensive sRNA-seq datasets.

**Database URL**: https://www.nipgr.ac.in/athisomiRDB

## Introduction

RNA-sequencing (RNA-seq) technologies have revolutionized the identification of non-coding RNAs (ncRNAs) [1]. Among these, miRNAs have emerged as extensively researched entities linked to regulatory roles in diverse biological processes and development in plants [2, 3]. Studies reveal that isomiRs, distinct miRNA isoforms with varying sequences and expression patterns, contribute to a dynamic miRNAome and influence the coding–ncRNA regulatory network [4–8]. Recently, much emphasis has been placed on the discovery of these variants to unravel their functions [9–13]. Indeed, the role of isomiRs has been demonstrated to be crucial in regulating processes at the post-transcriptional level, emphasizing their pivotal significance in biological systems within the broader context of miRNA research [14, 15].

In the past decade, miRNA researchers have harnessed deep sequencing techniques to explore the sequence and abundance of miRNAs. Contrary to the conventional belief that each pre-miRNA arm yields a singular consequential mature miRNA (the ‘reference’ miRNA), the discovery of isomiRs challenges this paradigm. Originally focused on canonical miRNAs documented in the miRBase database [16], recent investigations have brought attention to changes in both the length and sequence of miRNAs [8, 12, 17, 18], underscoring the importance of examining isomiRs. Small RNA-seq technologies, coupled with relevant computational tools, have unveiled a plethora of miRNA variants. These variants encompass heterogeneous 3′ or 5′ ends, differing lengths, and sequences originating from a single precursor miRNA. In addition, nontemplated additions by nucleotidyltransferases, such as noncanonical terminal uridylyltransferases and PolyA Polymerases, also comprise isomiRs [2, 19]. Growing evidence indicates that these modifications can impact miRNA sequence stability, confer different targets compared to the canonical form, or influence miRNA subcellular compartmentalization [4, 8, 20]. However, the biological significance of these observations remains under discussion.

Investigating isomiRs within existing databases poses challenges due to variations in both sequence and abundance, further complicated by the reliance on reference miRNA databases [21, 22]. Certain isomiRs dominate specific miRNAs; however, their low abundances, which are often near sequencing noise levels, can lead to oversight, resulting in prevalent mapping tools failing to detect these low-frequency isomiRs [23, 24]. Despite these challenges, a recent study underscored the efficacy of small RNA-seq methods in accurately capturing the diverse landscape of miRNAs without introducing significant errors, such as false isomiRs [25]. Even with this advantage, systematically collected and annotated isomiR information remains limited in available databases. Numerous isomiR databases offer sequence and expression data; however, comprehensive details on isomiR expression are provided by only a few. Despite the increasing availability of publicly accessible small RNA-seq (sRNA-seq) samples, several resources furnish limited data with insufficient consideration for target diversity, especially concerning terminal modifications and nontemplated addition variants of miRNAs. Given these challenges, a clear need exists for a comprehensive resource that compiles extensive miRNA profiles at isoform resolution, significantly benefiting the miRNA research community by addressing current limitations in available databases.

To this end, we have developed a database athisomiRDB to support and facilitate ongoing isomiR research in *Arabidopsis thaliana*. It offers a comprehensive collection of isomiRs across a large set of *A. thaliana* samples, containing 20 764 isomiRs with expression profiles across diverse samples, each accompanied by curated metadata. Its user-friendly web interface enables efficient exploration, access to annotations, and functional analyses for insights into the effects of isomiRs on their targets. This resource goes beyond the ‘one-locus-one-miRNA paradigm’ and serves as a valuable tool for identifying candidate functional isomiRs, fostering further experimental studies in *A. thaliana*.

## Materials and methods

### Data collection, processing, and analysis

**Figure 1.**
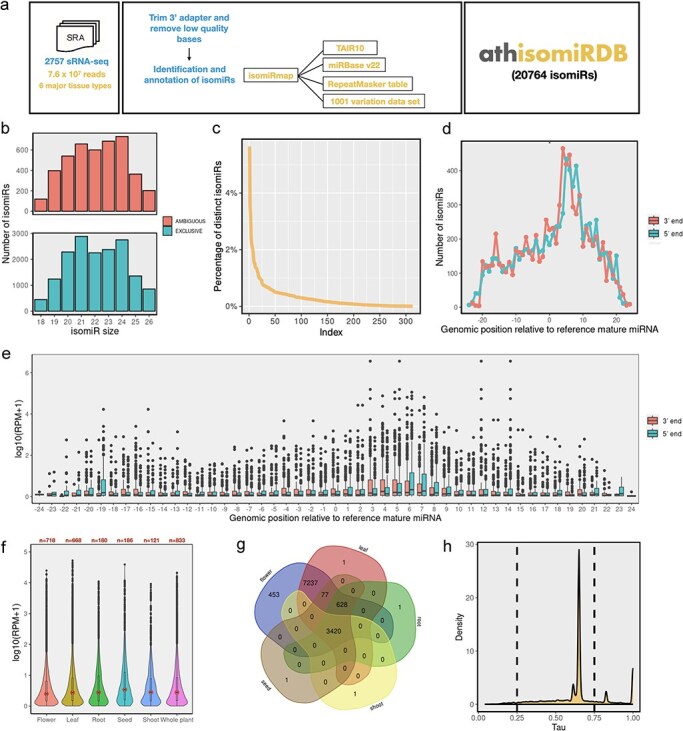
Overview of the annotation pipeline and summary of annotated isomiRs. (a) A total of 2757 publicly available *Arabidopsis* sRNA-seq libraries were collected from NCBI SRA databases and processed with a unified isomiRmap pipeline. All the isomiR-related information can be accessed via keyword-based searching on the athisomiRDB website. (b) Boxplots depict genomic instances of isomiRs (18–26 nts) from miRNA precursors in miRBase. (c) Count of unique isomiRs associated with each locus. (d) Distribution of the number of isomiR endpoints at each genomic position relative to the reference mature miRNA coordinate. (e) Relative expression of the isomiRs due to shifts in the 5ʹ or 3ʹ end at each genomic position relative to the reference mature miRNA coordinate. (f) IsomiR expression across categorized tissue types. (g) Number of unique and distinct isomiRs detected across major tissue types. (h) Frequency density plot of annotated isomiRs using the *τ* metric.

We searched sRNA-seq data of *Arabidopsis* in the National Center for Biotechnology Information (NCBI) Sequence Read Archive (SRA) database until July 2022, utilizing relevant keywords. A total of 2757 datasets were compiled (Supplementary Table S1); the data collection, processing, and database construction pipeline are detailed in Fig. 1. We applied ‘fastq-dump’ from the SRA Toolkit (v3.0.1) (https://github.com/ncbi/sra-tools) to convert raw data to FASTQ format. The fastp (v0.19.8) [26] tool was utilized to predict and trim 3′ adapter sequences if they were not provided. The isomiRs were identified, and expression data were profiled using isoMiRmap [22], a tool employing a brute-force approach and utilizing a ‘k-mer lookup table’ to catalog all wild-type isomiRs specified as either exclusive or ambiguous based on their presence in miRNA space. To avoid missing isomiRs that extend beyond the boundaries defined by the nominal precursor sequence in miRBase [16], we extended the miRNA arms by 6 nucleotides (nt) from the miRNA precursor sequence to also annotate boundary-straddling isomiRs. We generated a list of all possible unique 18–26 nt(inclusive) substrings that can be derived from each of these hairpin sequences. This list forms the first column of the k-mer exclusivity table, and tagging the isomiRs as exclusive or ambiguous generates the second column. Together, these columns represent all potential wild-type isomiR sequences that isoMiRmap is capable of profiling. A repeats table generated using transposable elements (TEs) annotated on the *Arabidopsis* genome assembly TAIR10 was applied, with TEs predicted using the Extensive *de novo* TE Annotator (v1.8.4) [27] with default settings. For each isomiR listed in the exclusivity k-mer table, we conducted an exhaustive search to identify its presence in the corresponding repeat class and capture all potential overlaps. Additionally, to identify isomiRs from common genetic variations cataloged in release 36 (June 2017) of the latest 1001 genetic variation dataset [28], we created a single nucleotide polymorphism (SNP) lookup table using the isoMiRmap tool. All the mapping tables used are available at https://github.com/skbinfo/athisomiRDB. Furthermore, we calculated the tissue specificity in terms of *τ*-values for each isomiR using expression data, following the formula described in Ludwig *et al*. [29].

### Prediction of isomiR targets

We predicted isomiR targets using psRNAtarget [30] with parameters based on Schema v2 (2017 release), except that the number of top targets was set to 10.

### Determining overlap between isomiR loci and multi-omics association data

We applied association identification to establish links between isomiRs and specific phenotypes or biological processes. Data, including expression Quantitative Trait Loci (eQTL), expression-methylation Quantitative Trait Loci, Genome-Wide Association Studies (GWAS), Transcriptome-Wide Association Studies, and Epigenome-Wide Association Studies, were obtained from *A. thaliana* multi-omics association database (AtMAD) [31]. The overlap between these features and different isomiR loci was determined using IntersectBed [32]. If any of this multi-omics data colocated with a particular isomiR, that isomiR was annotated for the associated phenotypes or biological processes as a predicted association.

### Database and website implementation

The athisomiRDB is implemented using Apache HTTP Server (v2.4.6), PHP (v7.3.3), and MySQL (v8.0.15) on a CentOS 7 Linux server. The back-end development involves JavaScript (v1.8.0) and PHP (v7.1), with data processing handled by MySQL (v8.0.15). Additionally, integration with BLAST (v2.11.0) enables online sequence similarity searches. The database incorporates interactive diagrams through Plotly libraries (https://plotly.com/). It is tested and supported on popular browsers such as Google Chrome, Firefox, and Safari.

## Results

### The transcriptional landscape of isomiR in *A. thaliana*

In this study, we processed 2757 *Arabidopsis* sRNA-seq datasets (7.59 × 10^7^ sequencing reads) using the isomiRMap tool for consistent analysis, as depicted in Fig. 1a. We considered 326 miRNA precursors from Release 20 of miRBase [16], defining an ‘active window’ for each miRNA hairpin arm. This window extended 6 nt beyond the boundaries of the ‘reference’ precursor listed in miRBase, retaining lengths from 18 to 26 nt inclusive. The approach allowed for the identification of various isomiR types, including 5ʹ-isomiRs, 3ʹ-isomiRs, 5ʹ- and 3ʹ-mixed isomiRs, 3ʹ nontemplated post-transcriptional additions concerning miRBase mature miRNA, and those with known SNPs from the 1001 Genomes Project [27]. The identified isomiRs encompass ‘wild-type’ isomiRs existing exclusively within the miRNA space and those found outside the miRNA space (ambiguous) in the *A. thaliana* (TAIR10) genome. Figure 1b illustrates ambiguous and exclusive isomiRs for each size [17–25], present at multiple genomic locations beyond the defined miRNA space. Subsequently, we identified 313 hairpins producing 20 764 distinct isomiRs (Supplementary Table S2). On average, each locus produces ∼70 distinct isomiRs, with significant variation depicted in Fig. 1c. Further examination of family-wise isomiRs revealed that out of 211 families, 146 have >20 isomiRs (Supplementary Table S3). In the subsequent analysis, our aim was to ascertain whether the observed isomiR variants displayed specific preferences in their 5′ or 3′ termini. In this regard, we investigated the distribution of isomiR termini relative to those of the corresponding miRBase entries (Fig. 1d). Among the 8057 unique isomiRs identified to overlap with reference mature miRNA, there is a notable prevalence of both 5ʹ and 3ʹ isomiR variations (∼97%), with only a few lacking variations at their ends. Moreover, there is no predominant size regarding the reference miRNA for single-end variation, indicating that the diversity observed in isomiRs largely stems from simultaneous variations in both the 5ʹ and 3ʹ termini. Additionally, within our compilation of isomiRs featuring potentially extended 3′ additions, incorporating one or more instances of A, C, G, or U, the two most prevalent types are uridylation (35.85%) and adenylation (27.7%) (Supplementary Figure S1).

We explored the variation in isomiR abundance concerning the position of each isomiR termini in relation to the reference miRNA. The average expression of each isomiR in detected samples was calculated, and the results were plotted against the deviation from the reference’s 5′ or 3′ termini (Fig. 1e). Notably, for the 5′ terminus, there was a consistent reduction in isomiR abundance, by up to an order of magnitude, as the distance from the reference 5′ position increased. In contrast, the abundance of isomiRs with termini ending up to 20 nt away (either −20 or +20) from the reference 3′ position remained relatively unchanged compared to that of the reference.

Our analysis revealed a wide range of isomiR expression levels (Supplementary Figure S2-A), with the majority showing low to moderate expression [log10(RPM + 1) values of <2] and a subset exhibiting high expression levels [log10(RPM + 1) values of >4]. Uniform Manifold Approximation and Projection analysis (Supplementary Figure S2-B) illustrated clustering of samples based on tissue type, with notable overlap between flower and leaf samples. Tissue-specific expression analysis (Fig. 1f) demonstrated varying patterns across plant organs. Seed tissue exhibited the highest median expression levels, followed by root and shoot tissues. Flower and leaf tissues showed lower median expression but a wider range, including some highly expressed outliers. Set analysis of isomiR presence across tissues (Fig. 1g) identified ∼16% of isomiRs common to all five tissue types, with root tissue showing the highest number of tissue-specific isomiRs (7337). Flowers were noted for having the highest number of unique isomiRs, with ∼450 not identified in other tissues. The correlation heatmap (Supplementary Figure S2-C) indicated high overall similarity in isomiR expression across tissues, with Pearson’s correlation coefficients ranging from 0.77 to 0.88. The highest correlation (0.88) was observed between leaf and flower tissues. Moreover, our data revealed a dichotomy in isomiR expression patterns; some were ubiquitously expressed, while others were exclusive to specific tissues, as illustrated in Fig. 1h. The distribution of *τ*-values (Fig. 1h), a measure of tissue specificity, revealed a bimodal pattern with peaks near 0 and 1. This indicates that while many isomiRs are broadly expressed across tissues (*τ* near 0), there is also a substantial population of tissue-specific isomiRs (*τ* near 1), highlighting the existence of tissue-specific isomiR signatures that could be crucial for specialized functions.

In this study, we profiled 2866 variant-containing isomiRs (Supplementary Table S4) in addition to wild-type isomiRs, as these variants have the potential to alter isomiR function or exhibit tissue- or developmental stage-specific expression. We identified a total of 4969 intersections (Supplementary Table S5) between isomiRs and repeat regions in *A. thaliana*, with the majority classified as DNA repeats (3024). Significant contributions were also observed from RC/Helitron (830), LTR/Gypsy (233), and DNA/MuDR (212). Other classifications included DNA/HAT (61), DNA/Helitron (109), and several minor categories such as DNA/Harbinger, LINE/L1, and LTR/Copia. Notably, 381 intersections remained unassigned, indicating areas for further investigation. Furthermore, we predicted a total of 201 789 targets across identified isomiRs (Supplementary Table S6), highlighting their extensive regulatory potential across various biological processes in *Arabidopsis*. Lastly, we identified a total of 2666 isomiR-QTLs (Supplementary Table S7) based on overlap with various QTL categories, including 89 *cis*-eQTLs, 38 *trans*-eQTLs, 1593 environment-related eQTLs, 332 GWAS-related eQTLs, 272 environment-associated eQTLs, and 342 ePathway-associated eQTLs.

Overall, the extensive isomiR catalog compiled in this study, along with these findings, emphasizes that the reference mature miRNA in miRBase is just one of several products emerging from the corresponding hairpin locus, emphasizing the complexity of the isomiR landscape in *A. thaliana*. All isomiR data have been systematically organized and are freely accessible at https://www.nipgr.ac.in/athisomiRDB.

### Functional sections of athisomiRDB

**Figure 2.**
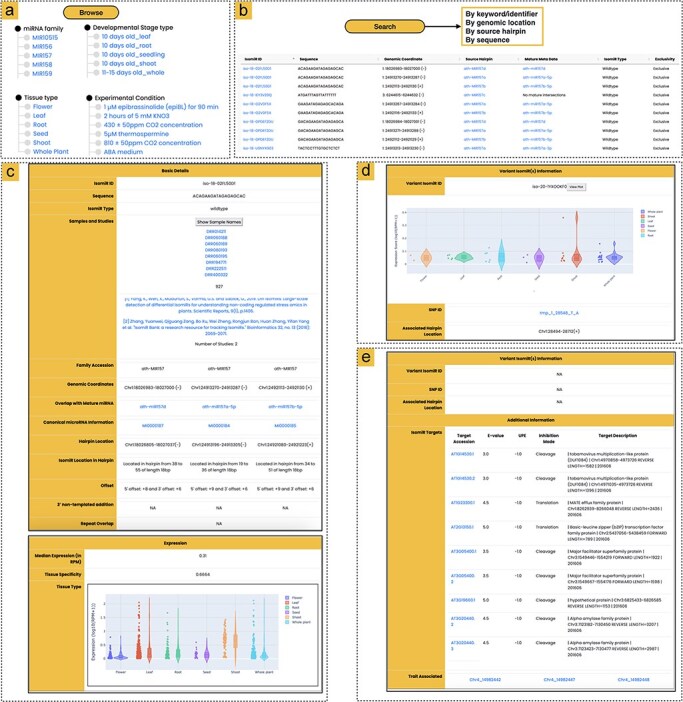
Overview of the athisomiRDB web interface features. (a) Browsing options for isomiR entries based on miRNA family, tissue type, developmental stage, or genotype. (b) Summary of available search engines for querying isomiR entries, with an example snapshot showing subset results in response to the keyword search ‘MIR157’. (c) Snapshots of isomiR iso-18-02FL50D1 from precursors MIR157d, MIR157a-5p, and MIR157b-5p, which were detected in 927 samples and two previously reported studies, along with expression information. (d) Variant isomiR information section presenting isomiRs with SNPs, such as iso-20-1YXOOKF0 from ath-MIR838, featuring a T > A alteration compared to the wild-type isomiR iso-20-9YXOOKF0. Details include the relevant SNP ID and expression profile of the variant isomiR. (e) Snapshot of iso-24-IP8E9Q9EK0, providing details on potential targets and associated SNPs linked to specific phenotypes. The isomiR is associated with three SNPs related to the flowering trait, identified from the AtMAD.

The athisomiRDB is designed for user convenience, offering easy access to the isomiR collection through search engines and a straightforward navigation experience with shortcuts and layered webpages (Fig. 2). In contrast to other publicly available isomiR databases like Plant isomiR Atlas [17], IsomiR Bank [13], and Diff isomiRs [12], athisomiRDB has utilized a larger number of sRNA-seq datasets, annotating a greater number of isomiR loci with reference to miRBase (Supplementary Table S8). Users are provided with the flexibility to download data in bulk according to their preferences. The functions of athisomiRDB are described in the sections below.

#### Browse

The ‘Browse’ interface facilitates a systematic exploration of isomiRs, organized by miRNA families (289), major tissue types [6], developmental stages (142), experimental conditions (68), and genotypes (326) (Fig. 2a). Moving to the next level of browsing, users encounter a summary of all isomiRs within a specific browse option. This summary includes key information such as the isomiR ID, sequence data, genomic location, overlap with the reference mature miRNA, isomiR type, source hairpin, and isomiR exclusivity. The isomiR ID serves as a unique identifier, known as a ‘license plate’ (https://cm.jefferson.edu/LicensePlates/help/about.jsp), ensuring consistency unaffected by future genomic changes or modifications in the miRBase labeling scheme. Additionally, beyond these fundamental details, isomiRs are classified as either ambiguous or exclusive, recognizing the potential origin of the isomiR from a genomic location beyond annotated hairpins. Furthermore, each isomiR miRNA entry, when clicked, seamlessly directs users to a detailed information page.

#### Search

Users can efficiently navigate the entire athisomiRDB using multiple search engines (Fig. 2b). The keyword or miRNA/isomiR identifier search engine enables users to conduct comprehensive searches across all fields of the database. Search results are presented in a list of relevant isomiRs, and users can conduct searches based on isomiR sequence, miRNA family name, or isomiR source (e.g. miRNA name, identifier, and tissue). For the convenience of quickly searching for miRNA information such as genome coordinates and source miRNA hairpin, athisomiRDB provides corresponding search engines. The platform features a BLAST-based sequence similarity search tool [33], accessible through a web interface. Users can submit their own sequences and customize the search parameters, such as the scoring matrix and e-value threshold, to tailor the analysis to their specific needs. Lastly, a search engine was built in to search for variant-containing isomiRs with reference to a selected precursor miRNA. This search enables users to obtain information about each variant isomiR, including its sequence, expression, parent mature miRNA, and precursor origin. Users can also access SNP variant isomiRs with identifiers linked to the Ensembl Plants database [34]. This option ensures efficient retrieval of information for 2866 variant isomiRs from the athisomiRDB database, along with their expression information.

#### Annotation details page

Upon clicking each isomiR ID, users are directed to a detailed information page divided into four sections: (i) basic details, (ii) expression information, (iii) variant isomiR information, and (iv) additional information. The breakdown of each section is outlined below.

##### Basic details

In the ‘Basic details’ section, users can explore vital information, including sequence ID, isomiR sequence, isomiR type, family accession, genomic coordinates, and the overlap with reference miRNA, which is linked to miRBase for additional details on mature miRNA. This section also includes canonical miRNA information, linked to the corresponding hairpin details in miRBase [16], and specifies the isomiR’s location within the hairpin (Fig. 2c). The athisomiRDB furnishes users with information about the identity of all miRNA precursors that contain a specific isomiR, along with details on the location within each precursor where the isomiR can be found. Additionally, the section delves into offsets, highlighting nucleotide sequence variability at the 5′ and 3′ ends concerning mature miRNAs. Furthermore, details on 3′ nontemplated additions are available, encompassing occurrences of A, C, G, or U, as well as potentially elongated sequences of 3′ additions. The conclusion of this section emphasizes whether a specific isomiR is present within a recognized repeat element. Users are also provided with information on repeats and additional links for exploring repeat ontology. In instances where an isomiR overlaps with multiple repeat/transposon classes, athisomiRDB offers a comprehensive listing of each class.

##### Expression information

The expression profiles of each isomiR across major tissue types are displayed in the expression section, visualized through a violin plot (Fig. 2d). The *τ*-value provided by the database quantifies the selective expression of each isomiR across six tissue types. A higher *τ*-value indicates greater tissue specificity, suggesting potential functional roles in those tissues, while a lower *τ*-value reflects more uniform expression. This metric aids users in exploring the functional implications of isomiRs in tissue contexts.

##### Information on variant-containing isomiRs

The IsomiRs containing SNP variants are documented alongside their respective wild-type counterparts in relation to the 1001 Genomes Project [28] in this section. The information includes the isomiR sequence with the SNP variant, the corresponding wild-type isomiR sequence, normalized abundances, and the SNP ID associated with the SNP-containing isomiR (Fig. 2e). Users also have the option to explore the SNP ID linked to the Ensembl Plants database [34]. In cases where multiple SNP-containing isomiR sequences are present, all are displayed. Unlike wild-type isomiRs, variant-containing isomiRs do not include additional data related to transposon overlaps or miRNA-space exclusivity information.

##### Additional information

The additional information section in athisomiRDB provides valuable insights into potential targets of the corresponding wild-type isomiR and offers details on multi-omic associations with the isomiR locus (Fig. 2e). Users can explore the top 10 predicted targets for isomiRs in this section to gain an understanding of their potential connections to downstream pathway functions. Complementing this, the isomiR-trait associations section enhances the exploration of SNPs that impact isomiRs, particularly those coinciding with established trait-associated loci. This is supported by experimental evidence available through the multi-omic associations linked to the AtMAD database [31].

### Case Study 1: several isomiRs associate with Argonaute complexes

A defining characteristic of miRNAs lies in their connection with the AGO (Argonaute) silencing complex, which serves as a mediator of regulatory effects on target genes [2, 35–37]. Despite this, the intricate landscape of isomiR populations and the diversity within the AGO family pose crucial inquiries regarding the mechanisms governing the selective sorting of isomiRs into distinct AGOs. Consequently, in this case study, we investigated the interactions between isomiR populations and the AGO complex, specifically focusing on their sorting into different AGOs.

**Figure 3.**
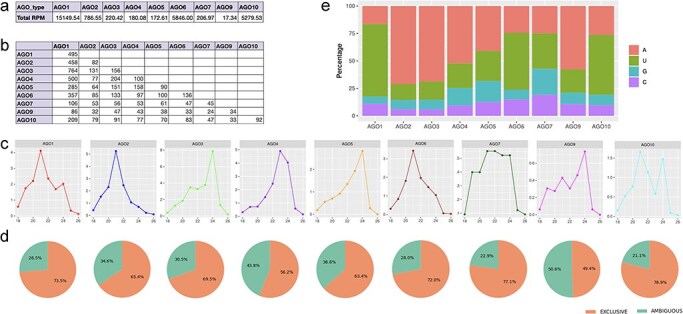
Characteristics of isomiRs bound to each AGO complex. (a) Summary of normalized expression for all isomiRs bound to each AGO complex. (b) Comparison of small RNA populations bound by the nine AGO complexes, with numbers indicating unique overlapping isomiRs. (c) Size distribution of isomiRs bound by the nine AGO complexes. (d) Pie charts summarizing exclusive and ambiguous isomiRs for these complexes. (e) The relative frequency of each 5′ terminal nucleotide bound by each AGO complex.

Using isomiRs available at athisomiRDB, we examined 28 public AGO-RIP (Argonaute-RNA Immunoprecipitation) datasets (Supplementary Table S9). We analyzed normalized read counts from AGO-RIP sRNA-seq libraries for each AGO complex, except AGO8, as it was not available. Out of 20 764 isomiRs, 1952 were identified as being associated with any AGO complex (Supplementary Table S6). In alignment with the recognized role of AGO1-bound miRNA [35], most reads exhibited evidence of AGO1 association with isomiRs. AGO-RIP expression data suggest a redundant function of AGO1 with other complexes (refer to Fig. 3a), and notably, AGO2, AGO6, and AGO10 also exhibit a strong association with isomiRs (Supplementary Table S10). Moreover, diverse isomiRs associated with AGO3 imply functional divergence, albeit not as pronounced as with the other AGOs mentioned earlier (Supplementary Table S10). It is noteworthy that AGO3 has an established role in generating 24-nt short interfering RNAs (siRNAs), as reported previously [38]. Furthermore, to comprehend the relationship between isomiRs present in distinct AGO complexes, we conducted a comparative analysis of isomiR sequences among the nine AGO datasets. The results exhibit a limited number of shared isomiRs between datasets, suggesting that distinct AGO complexes recruit specific subsets of isomiRs. This observation implies a loading bias, with discernible preferences for isomiRs among different AGO complexes (Fig. 3b).

Subsequently, we examined the influence of isomiR sequence length on various AGO types, revealing a prevalence of 21- and 24-nt isomiRs. Specifically, AGO1, AGO10, AGO2, AGO7, and AGO6 displayed dominance in 21-nt isomiRs, while AGO3, AGO10, AGO7, and AGO9 exhibited a preference for 24-nt isomiRs (Fig. 3c). However, drawing definitive conclusions regarding the significance of isomiR length in AGO sorting necessitates more extensive datasets encompassing a broader array of AGO complexes than those considered in this analysis. Notably, AGO8 was excluded due to unavailability, and certain AGO complexes lacked libraries or had insufficient deep sequencing, as evident from the total number of reads mapped. Additionally, it is crucial to highlight the existence of ambiguous isomiRs across AGO types, suggesting the potential origin of isomiR sequences from alternative genomic loci, such as siRNA, tRNA-derived small RNAs, or rRNA-derived small RNAs (Fig. 3d). Furthermore, the data depicted in Fig. 3e reveal a pronounced bias for 5′ terminal nucleotides in sequences associated with each AGO complex. Notably, no specific nucleotide preference is discerned at other positions within isomiRs linked to AGO complexes, irrespective of isomiR size (Supplementary Table S7). The selective recruitment of isomiRs by AGO complexes, particularly those with distinct 5′ terminal nucleotides, suggests varying binding affinities, consistent with previous reports [39, 40]. Consequently, altering the 5′ terminal nucleotide of an isomiR may influence its biological activity. Moreover, the tissue-specific and developmental expression patterns of AGO complexes likely contribute to the preferential recruitment of isomiRs sharing similar expression profiles. These findings point toward the general properties of AGO complexes in compartmentalizing miRNA regulatory pathways, highlighting their role in shaping the intricacies of miRNA function. Overall, the AGO-RIP datasets in this case study support the link between athisomiRDB isomiRs and the AGO silencing complex, indicating potential roles in the RNA interference pathway.

### Case Study 2: naturally occurring isoforms of miR775 may cooperatively target shared biological pathways

**Figure 4.**
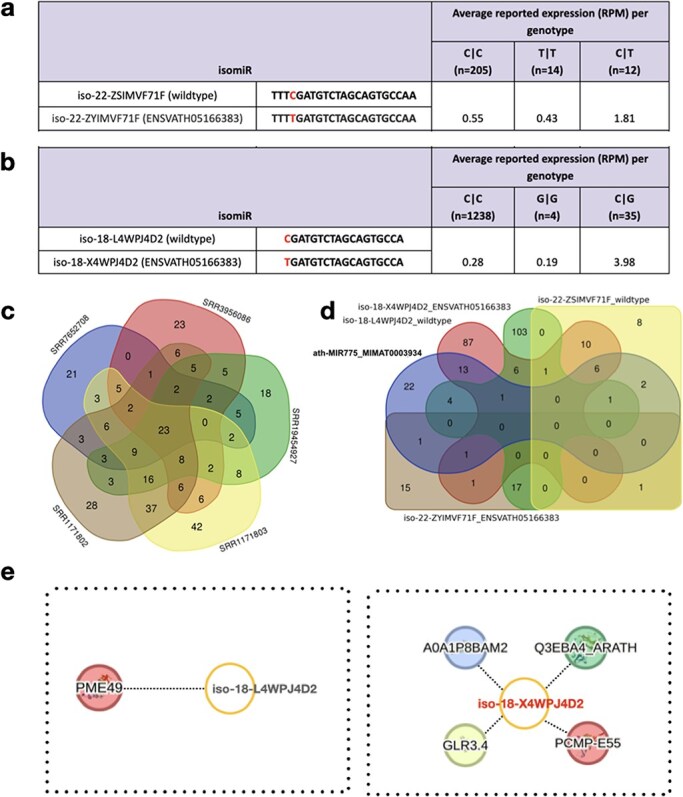
Wild-type and variant-containing isomiRs and their targets. (a-b) Two wild-type isomiRs and their corresponding variant-containing isomiRs (based on known SNPs from the 1001 Genomes Project data), along with their respective expression values in athisomiRDB across samples. Below each genotype, the number of samples in which the genotype is present is indicated. (c) Venn diagram depicting the overlap between the target genes supported by five PARE-seq datasets. (d) A five-way Venn diagram depicting the overlap between the target genes of canonical miRNA and the other four isomiRs, as indicated in (a-b). (e) IsomiR–mRNA target network of iso-18-L4WPJ4D2 (wild type) and iso-18-X4WPJ4D2 (ENSVATH05166383).

In recent studies, the critical involvement of miR775 in *A. thaliana* was elucidated, specifically in the post-transcriptional silencing of Galactosyl Transferase (GALT) [41–43]. To assess the impact of SNPs within wild-type isomiRs of miR775, we examined the presence of both wild-type and variant isomiRs in athisomiRDB. We verified this through the analysis of two wild-type isomiRs, namely iso-22-ZSIMVF71F and iso-18-L4WPJ4D2 of MIR775, obtained from athisomiRDB (Supplementary Table S11). The results of this examination, presented in Fig. 4a and b and Supplementary Table S11, revealed distinct genotypes (C|C, T|T, and C|T) for the 22-nt isomiR and (C|C, G|G, and C|G) for the 18-nt isomiR in relation to the canonical miR775. These data highlight the presence and expression levels of these wild-type and variant isomiRs, demonstrating the representation of all genotypes in diverse *Arabidopsis* samples within athisomiRDB.

To decipher the biological role of miRNAs involved in the translational machinery, with a specific focus on the repression of mRNA targets, we investigated the combined athisomiRDB target data and Parallel Analysis of RNA Ends (PARE)-seq data analyzed using the Cleaveland tool [44] (Supplementary Table S12). As expected from PARE-seq, we only considered 0–2 category targets, of which numerous category 2 targets yielded a total of 691 targets across the five PARE-seq libraries considered in this case (Fig. 4c and d). Additionally, upon examining recognition sites within select transcripts, our analysis revealed that some isomiRs exhibit the capability to target the same site as the canonical miRNA, albeit with slight nucleotide variations (Supplementary Table S13). If we consider the mode of targeting as only inhibiting and not cleaving, the regulation appears to be nonrandom, showing substantial overlap in functional mRNA networks suppressed by both canonical miRNAs and their isomiRs. However, we limited the search to the highest confidence (‘Category 0–1’) targets to identify the most abundant PARE-seq-supported targets. This enabled us to find seven distinct targets other than the GALT, as reported previously for reference/canonical miR775 (Supplementary Figure S3; Fig. 4e). This example of targets cleaved by isomiRs but not by their canonical miRNA, derived from applying strict parameters, highlights the importance of studying both reference mature miRNAs and isomiRs for a comprehensive understanding of their functions. Thus, with this case study, we emphasize isomiRs as biologically relevant entities, acting as cooperative partners with canonical miRNAs to target functionally related gene pathways in *A. thaliana*.

## Conclusions and future directions

Extensively studied for their pivotal roles in various biological processes, miRNAs are predominantly investigated in their canonical form, with limited attention given to the multiple isomiRs, which often surpass the canonical miRNA in abundance and significance [10, 24, 45]. Hence, creating a public resource is crucial for future isomiR studies, given the diverse isomiRs that maintain close functional relationships. Toward this end, we have established a comprehensive annotated resource of *Arabidopsis* isomiRs, known as athisomiRDB. Our large-scale isomiR repository represents 20 764 isomiRs identified and annotated from a total of 2757 sRNA-seq datasets. The athisomiRDB platform provides users with comprehensive information on the miRNA precursors that contain the identified isomiR sequences. The database aims to offer an overview of both high- and low-abundance isomiRs across diverse samples, covering distinct tissues and capturing even the least expressed isoforms. Compared with current databases containing *Arabidopsis* isomiRs, athisomiRDB is more comprehensive in terms of covered samples, including features extracted from multi-omics data, such as expression profiles, genome variation of isomiR loci, association with phenotypes, and isomiR–mRNA interactions (Supplementary Table S8). In addition, the athisomiRDB platform includes information on the occurrences of isomiRs that have one or more 3ʹ nontemplated nucleotide additions, which are post-transcriptional modifications. In summary, athisomiRDB stands as the first database enabling multi-omics association analysis in *Arabidopsis*, facilitating the discovery of plant biological processes at different levels of evidence. We believe that the compiled data in athisomiRDB can be used to answer several research questions related to the expression of isomiRs. Future development of athisomiRDB will involve regular updates of newly discovered miRNAs and isomiRs, with a focus on validation information regarding isomiR processing by Dicer-like. Additionally, our goal is to gather diverse representatives, including multiple species, and compile numerous high-quality, unbiased datasets covering a comprehensive range of tissues, cell types, and developmental stages, while including more isomiR–phenotype associations. We look forward to suggestions from scientists worldwide to continually enhance and broaden the utility of athisomiRDB.

## Supplementary Material

baae115_Supp

## Data Availability

The athisomiRDB is accessible at https://www.nipgr.ac.in/athisomiRDB, and other essential IsoMiRmap lookup tables for annotating isomiRs of *A. thaliana* are available at https://github.com/skbinfo/athisomiRDB.
