# Supplementary figures and images for: athisomiRDB: A comprehensive database of Arabidopsis isomiRs
Source: Database (Oxford). 2024 Nov 8;2024:baae115. doi: 10.1093/database/baae115 (PMC11544919; doi:10.1093/database/baae115)

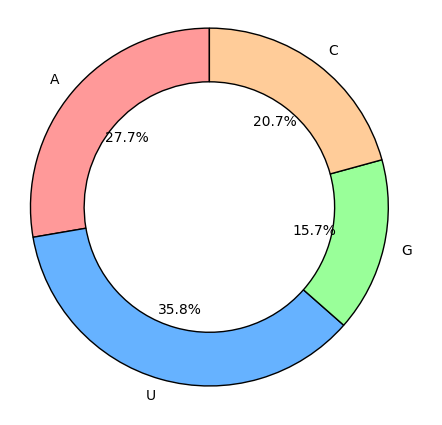

Supplement: baae115_Supp [file baae115_supp.zip › baae115_Supp/Supplementary Figure S1.tiff]

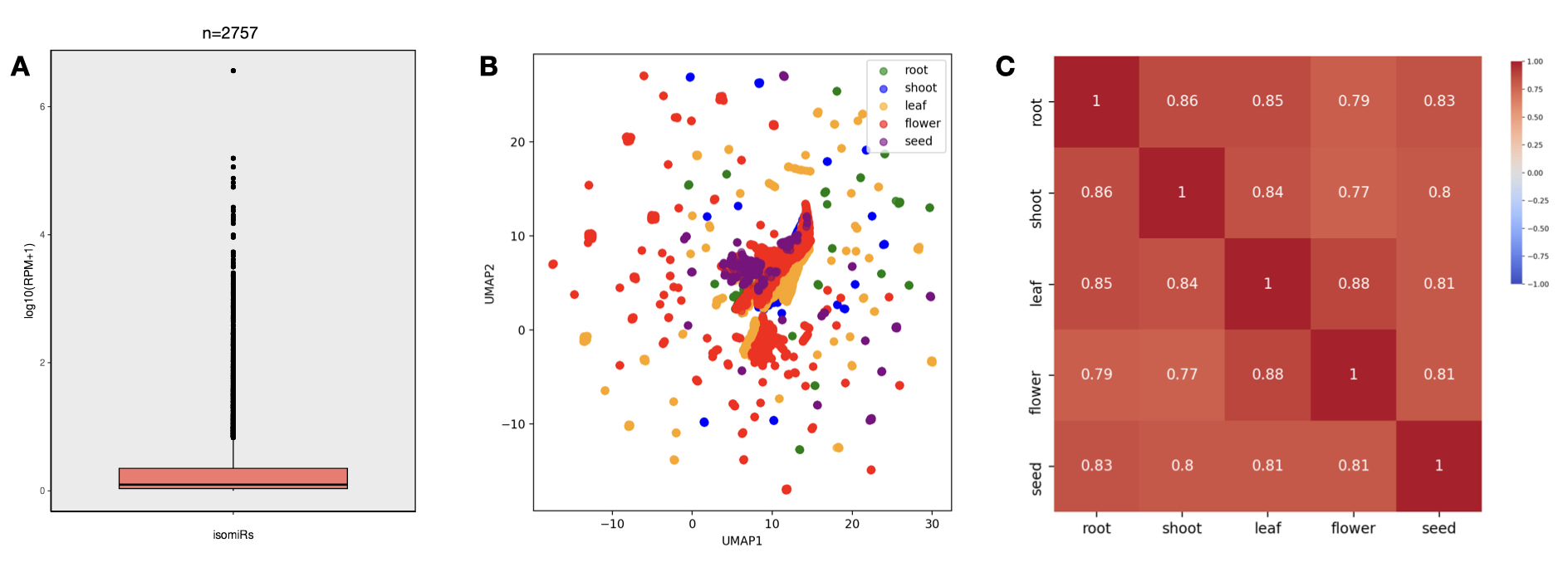

Supplement: baae115_Supp [file baae115_supp.zip › baae115_Supp/Supplementary Figure S2.tiff]

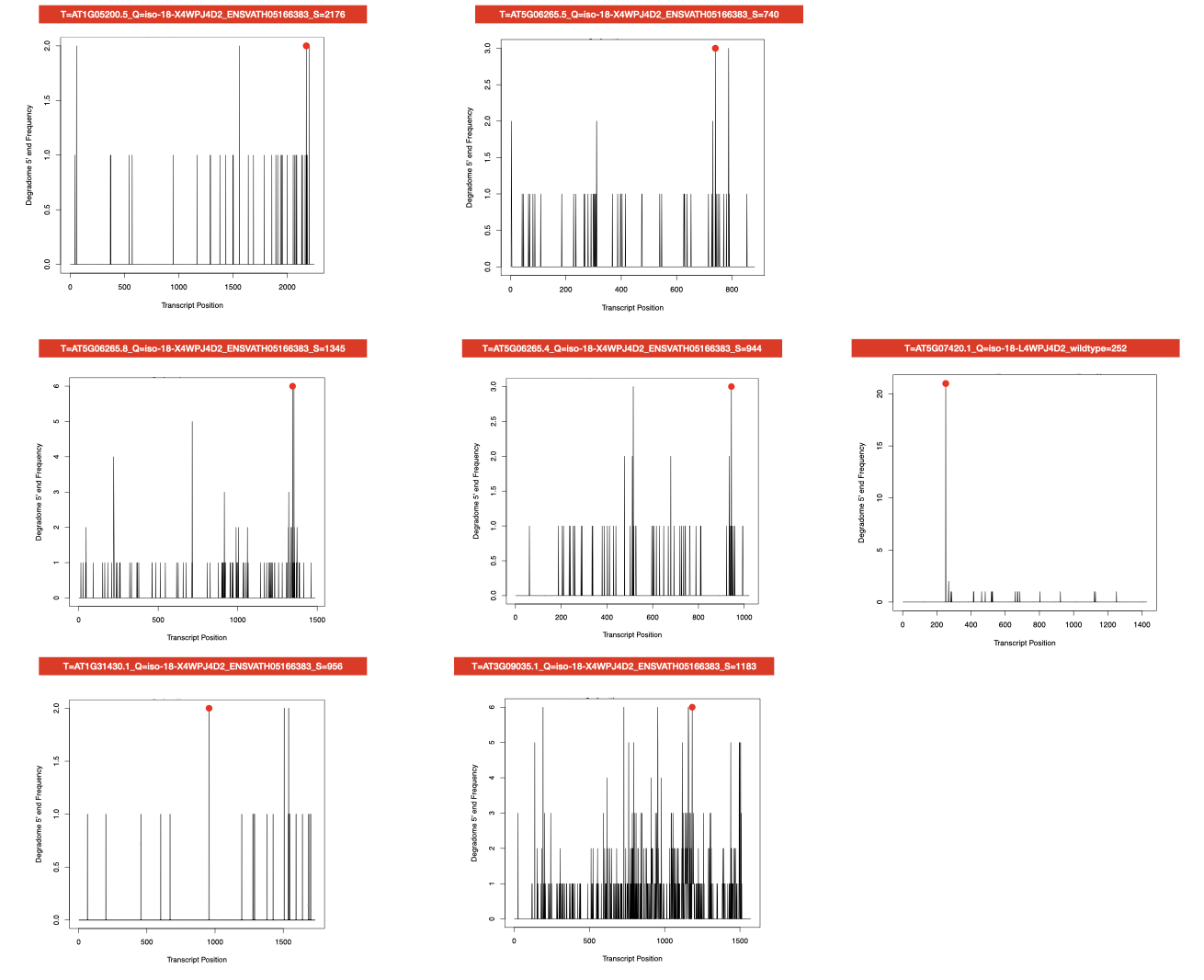

Supplement: baae115_Supp [file baae115_supp.zip › baae115_Supp/Supplementary Figure S3.tiff]
